# Mechanism of Structure and Property Evolution of ABS During Multiple Extrusion and Aging Degree Prediction via Image Recognition Technology

**DOI:** 10.3390/polym18111410

**Published:** 2026-06-05

**Authors:** Lin Su, Hongxing Wang, Haozhan Wu, Jianjun Yi, Hu Hui

**Affiliations:** School of Mechanical and Power Engineering, East China University of Science and Technology, Shanghai 200237, China

**Keywords:** ABS resin, multiple extrusion, image recognition, deep learning, thermo-oxidative degradation

## Abstract

The recycling of acrylonitrile-butadiene-styrene (ABS) is crucial for a circular plastics economy, but repeated extrusion induces degradation that limits its reuse. This study establishes a comprehensive structure-property evolution mechanism for ABS 757K over five extrusion cycles and develops a novel image-recognition model for aging degree prediction. Multi-faceted characterization revealed that chain scission, oxidation of the polybutadiene (PB) phase, and the formation of chromophores led to progressive embrittlement, yellowing, and reduced thermal-oxidative stability. A key finding from Energy Dispersive Spectroscopy (EDS) was the stability and homogeneous distribution of sulfur-based antioxidants, which underpin the material’s superior resistance to degradation by effectively scavenging free radicals, which function as effective free radical scavengers. This mechanism underpins the material’s superior resistance to thermo-oxidative degradation. Consequently, significant molecular weight reduction and property deterioration were delayed until later extrusion cycles. Furthermore, a deep learning model based on the DeepLabV3+ architecture was trained to predict extrusion history directly from scanning electron microscopy (SEM) images of impact-fractured surfaces. The model achieved an average prediction accuracy exceeding 96.5%. Remarkably, it demonstrated excellent generalizability, maintaining high accuracy on two unseen commercial ABS grades. This indicates that the micro-morphological evolution pathway is a universal fingerprint of thermo-mechanical aging in ABS. This work not only elucidates the multi-scale degradation mechanism of recycled ABS but also provides a rapid, non-destructive tool for intelligent quality assessment in plastic recycling streams, bridging advanced machine learning with practical sustainability challenges.

## 1. Introduction

Acrylonitrile-butadiene-styrene (ABS) terpolymer is a high-performance engineering thermoplastic valued for its outstanding toughness, good dimensional stability, and excellent processability [1]. These attributes have cemented its status as a material of choice across a diverse spectrum of industries, including automotive, electronics, and consumer goods. In the context of a growing global emphasis on sustainability and the circular economy, the mechanical recycling of post-consumer and post-industrial plastic waste has gained paramount importance [2,3,4]. For thermoplastics like ABS, recycling predominantly involves multiple cycles of melt processing, such as extrusion and injection moulding. However, it is well-established that repeated exposure to high temperatures and shear forces during these processes can induce significant thermo-mechanical and oxidative degradation [5,6]. This degradation manifests as chain scission, cross-linking, and changes in the composition of the polybutadiene rubber phase, leading to the irreversible deterioration of key mechanical properties (e.g., impact strength, tensile properties), discoloration (increased yellowing), and ultimately, a limitation on the material’s useful service life in subsequent applications [7,8,9]. Therefore, understanding and quantifying the degradation incurred during recycling is crucial for determining the limits and applications of recycled ABS.

Extensive research has been devoted to understanding the degradation mechanisms of polymers under thermo-oxidative conditions. For ABS, the unsaturated butadiene phase is particularly susceptible, with degradation pathways involving chain scission, cross-linking, and the formation of oxygen-containing functional groups. Recent studies have continued to investigate the thermo-mechanical degradation of ABS during multiple-extrusion cycles using FTIR, GPC, and mechanical testing [10], while machine learning approaches have also emerged for predicting polymer degradation states from spectroscopic data [11]. While traditional analytical techniques—including Fourier-transform infrared spectroscopy (FTIR) for chemical structure, differential scanning calorimetry (DSC) for thermal transitions, gel permeation chromatography (GPC) for molecular weight distribution, and rheological measurements for melt behaviour—have been instrumental in characterising these changes, a comprehensive and quantitative correlation between the evolution of micro-structural features and the resultant macro-scale performance across sequential processing cycles remains an area requiring deeper exploration [12,13,14,15,16,17]. Most studies focus on isolated properties or a limited number of processing cycles, failing to provide a holistic view of the progressive structure-property trajectory. Furthermore, the prediction of the extent of degradation, or “aging degree”—operationally defined in this work as the number of extrusion cycles experienced by the material, which correlates strongly with a composite index derived from molecular weight reduction, impact strength loss, yellowing, and morphological changes—often relies on these sophisticated laboratory techniques, which can be time-consuming, destructive, and require specialised instrumentation and expertise.

This gap highlights the need for innovative, rapid, and non-destructive evaluation methods that can bridge microstructural evolution with serviceability assessment. In recent years, advances in computer vision and machine learning (ML) have opened new frontiers in materials science [18,19,20,21,22,23]. Image recognition technology offers a powerful paradigm for extracting quantitative data from material micrographs, such as fracture surface morphology. The texture, roughness, and topographic features of an impact-fractured surface are direct visual fingerprints of the material’s internal structure and failure mechanisms, which are intrinsically linked to its processing history and degradation state [24,25,26,27].

Therefore, this study is designed to address these challenges by establishing a detailed mechanistic framework for the recycling-induced degradation of ABS and developing a novel, image-based predictive tool. The core objectives of this work are twofold: (1) to systematically investigate the mechanism of structure and property evolution of ABS resin subjected to multiple-extrusion cycles, and (2) to develop a robust aging degree prediction model based on image recognition technology. To achieve the first objective, a batch of virgin ABS will be processed through up to five consecutive extrusion passes. The evolution of molecular structure will be meticulously characterised at each stage using FTIR, DSC, rheology, EDS, and GPC. Concurrently, the corresponding macro-scale performance, including key mechanical properties (tensile, impact) and optical properties (yellowness/whiteness index), will be evaluated. The integration of these datasets will enable a comprehensive mechanistic discussion on the cause-effect relationships between chain-level alterations and bulk material behaviour.

The second, synergistic objective leverages the acquired knowledge and samples. The impact fracture surfaces of specimens from different extrusion cycles will be captured as high-resolution digital images. These images will serve as the input dataset for a machine learning model. Feature extraction algorithms will quantify the morphological descriptors of the fracture surfaces. Subsequently, supervised learning algorithms will be trained to establish a direct correlation between these image-derived features and the quantitatively defined “aging degree” of the material (derived from the structural and performance data). The ultimate goal is to create a technology for aging degree based on image recognition capable of accurately identifying an unknown ABS sample’s processing history and degradation state solely from its fracture morphology. This research not only deepens the fundamental understanding of ABS degradation but also proposes a practical, intelligent tool for quality control and lifecycle assessment in plastic recycling, contributing to the advancement of a smarter circular economy for polymers [28,29,30].

## 2. Experiment Section

### 2.1. Materials and Sample Preparation

Acrylonitrile butadiene styrene (ABS) 757K was produced by Zhenjiang Chimei Chemical Co., Ltd. (Zhenjiang, China). In this research, ABS of the same standard was always used, and no addition was blended to the ABS matrix.

**Figure 1:**
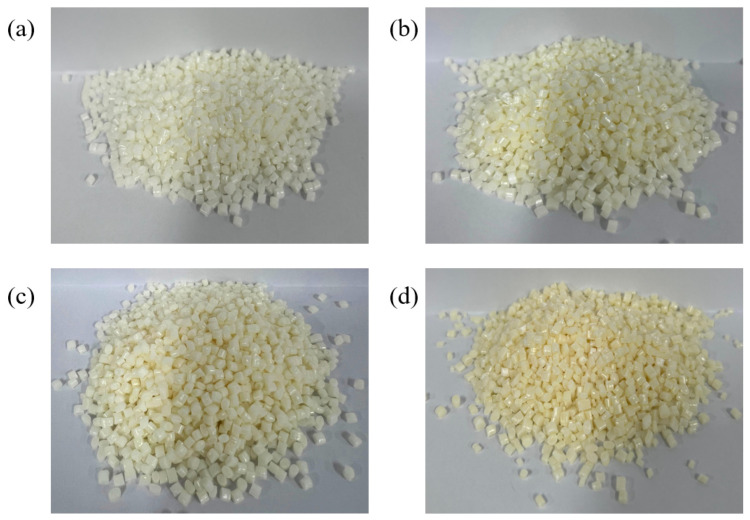
Photographs of (**a**) 757K, (**b**) 757K-1, (**c**)757K-3 and (**d**) 757K-5.

ABS pellets were used for molecular structure testing, while for mechanical testing, ABS specimens were prepared by compression molding in accordance with standard ISO 527-2:1996 and ISO 180:2023 [31,32]. The tested samples were crushed and extruded using an extruder under identical extrusion conditions. The samples subjected to 1, 3, and 5 extrusion cycles were designated as 757K-1, 757K-3, and 757K-5, respectively. Figure 1 is the virgin pellets and pellets after 1, 3, and 5 extrusion cycles. Cycles 2 and 4 were omitted because preliminary experiments indicated that the most substantial and mechanistically informative changes in molecular structure, mechanical properties, and morphology occurred at the 1st, 3rd, and 5th passes, while cycles 2 and 4 exhibited only incremental intermediate variations that did not alter the overall degradation trend. This spaced sampling strategy is widely adopted in multiple-extrusion studies to efficiently capture the degradation trajectory without unnecessary analytical burden.

The repetitive extrusion processing was performed using a co-rotating twin-screw extruder (SHJ-20, Nanjing Giant Machinery Co., Ltd., Nanjing, China) with a screw diameter of 21 mm and a length-to-diameter (L/D) ratio of 32. The screw configuration comprised standard conveying elements in the feed and transport zones, followed by a mixing section with two sets of 45° and 60° kneading blocks (total length 120 mm), and finally conveying elements for melt homogenization. The temperature profile along the eight barrel zones from the feed throat to the die was set as follows: Zone 1 (feed): 185 °C, Zone 2: 185 °C, Zone 3: 195 °C, Zone 4: 195 °C, Zone 5: 205 °C, Zone 6: 205 °C, Zone 7: 210 °C, and Zone 8 (die): 210 °C. The die temperature was also maintained at 210 °C. The screw rotation speed was maintained at 60 rpm, and the feed rate was controlled at 5 kg/h. Under these conditions, the average residence time was estimated to be 90–120 s, as verified by tracer experiments using 0.1 wt% carbon black. After extrusion, the melt strand was cooled in a water bath (length 1.5 m, tap water at 20–25 °C) and then air-dried. The strand was subsequently pelletized using a strand pelletizer (rotation speed ~300 rpm) to produce cylindrical pellets of approximately 3 mm length and 2 mm diameter. For each subsequent processing cycle, the obtained pellets were dried in a vacuum oven at 80 °C for 4 h under an absolute pressure of 0.08 MPa to remove residual moisture before being fed into the extruder again. The complete reprocessing sequence was virgin pellets → 1st extrusion → pelletizing → drying → 2nd extrusion → pelletizing → drying → 3rd extrusion → pelletizing → drying → 4th extrusion → pelletizing → drying → 5th extrusion. Samples for characterization (designated 757K-1, 757K-3, and 757K-5) were taken after the 1st, 3rd, and 5th passes, respectively. All processing parameters were kept identical across all cycles to isolate the effect of the thermo-mechanical history itself.

### 2.2. Characterization

#### 2.2.1. Fourier Transform Infrared (FTIR) Spectroscopy

Fourier transform infrared spectra were recorded on a Nicolet 560 (Nicolet Co., Madison, WI, USA) Fourier transform infrared spectrometer at wavelengths ranging from 400 to 4000 cm^−1^ and a resolution of 4 cm^−1^. Tests were conducted at 25 °C.

#### 2.2.2. Gel Permeation Chromatography (GPC)

The molecular weight of the ABS was measured by a Waters Breeze QS GPC tester (Waters Breeze, Milford, MA, USA) at 25 °C. The solvent used was tetrahydrofuran (THF).

#### 2.2.3. Scanning Electron Microscopy (SEM) and Energy Dispersive Spectroscopy (EDS)

A scanning electron microscope Nova NanoSEM 450 (Thermo Fisher, Waltham, MA, USA) equipped with an Oxford X-Max 80 EDS detector was used to observe the section morphology after impact property tests. The SEM images were acquired at an accelerating voltage of 2.5 kV, a working distance of 5 mm, and a probe current of 100 pA, using the in-lens secondary electron detector. EDS analysis was performed at an accelerating voltage of 15 kV, with a collection time of 10 min per map, a pixel resolution of 512 × 384, and an average count rate of approximately 2000 cps. All analyses were conducted on the same impact-fractured surfaces used for mechanical testing. To ensure representativeness, two different locations were selected for each sample.

#### 2.2.4. Differential Scanning Calorimetry (DSC)

A DSC 3+ (METTLER TOLEDO Co., Zurich, Switzerland) was used to conduct differential thermal analysis of the materials. The samples (about 4 mg) were chopped up and placed in an aluminum crucible. The samples were heated from 25 to 300 °C in an air atmosphere at a rate of 10 °C/min. The extended upper temperature was chosen to fully capture the exothermic degradation event.

#### 2.2.5. Analysis of Mechanical Properties

**Figure 2:**
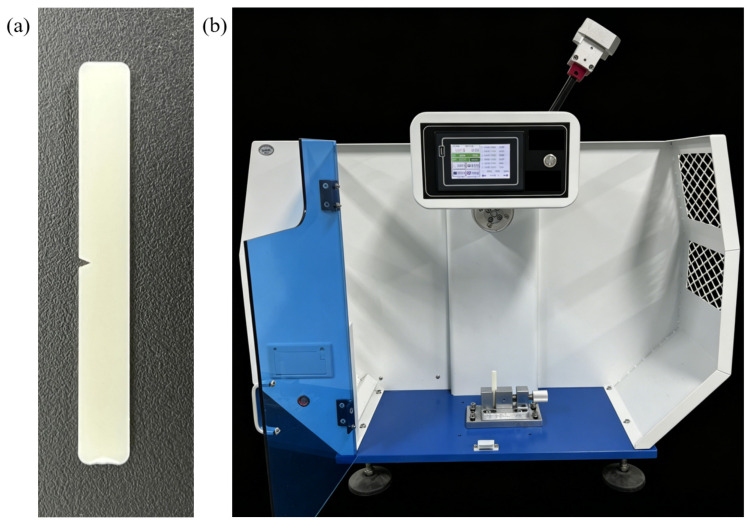
Photographs of (**a**) impact specimen and (**b**) impact testing setup.

The impact property of the sample was tested by a Cantilever beam impact testing machine (Chengde Jinjian Testing Instrument Co., Ltd., Chengde, China) according to ISO 180:2023 (Type 1 notched specimens). A photograph of the testing setup and impact specimen is shown in Figure 2. An Instron 865 universal material testing machine (Instron, Norwood, MA, USA) was used for tensile testing.

#### 2.2.6. Yellow Index (YI)

An automatic colorimeter NR60C (Shenzhen Sanenshidai Technology Co., Ltd., Shenzhen, China) was used to measure the yellow index.

#### 2.2.7. Melt Flow Rate (MFR)

The melt flow rate was measured by a CS-127 melt index tester (Custom Scientific Instrument, INC., Easton, PA, USA) with loads of 2.16 kg and 5 kg and the temperature at 230 °C. The flow rate ratio (FRR) is the ratio of MFR at 5 kg load to MFR at 2.16 kg load, which can characterize the molecular weight distribution of ABS melt.

#### 2.2.8. Image Recognition and Modeling Methodology

To achieve the objective of predicting the number of extrusion cycles experienced by ABS samples from their impact fracture surface morphology, a deep learning-based image recognition and modeling pipeline was designed and implemented in this study. The methodology primarily consists of four stages: image data acquisition, image preprocessing, feature extraction, and model construction and training.

(1) Image Data Acquisition

The original dataset comprised impact fracture surface morphology images of ABS samples subjected to different numbers of extrusion times (1, 3, and 5 times). High-resolution secondary electron images were acquired using a SEM under consistent operating conditions, including acceleration voltage, working distance, and signal acquisition mode, to ensure comparability in contrast and detail. A total of 576 sub-images were generated by cropping, all resized to a uniform resolution of 256 × 256 pixels. Each image was annotated with the corresponding extrusion time count of its ABS sample, thereby forming a labeled dataset requisite for supervised learning.

(2) Image Preprocessing

A series of preprocessing operations was applied to the raw SEM images prior to feature extraction to enhance training efficiency and model generalizability. First, image enhancement techniques, such as Contrast Limited Adaptive Histogram Equalization (CLAHE), were employed to improve the visual contrast of microstructural features. Subsequently, noise suppression algorithms, including non-local means denoising or median filtering, were applied to mitigate potential noise introduced during image acquisition while preserving crucial morphological boundaries. To improve model generalizability and mitigate overfitting, online data augmentation was applied exclusively to the training set. The augmentation pipeline included random horizontal flipping (probability 0.5), random vertical flipping (probability 0.5), random rotation within ±15°, and random scaling between 0.9 and 1.1. No augmentation was applied to the validation or test sets.

(3) Feature Extraction

This study employed the deep learning-based encoder–decoder architecture, DeepLabV3+, for automatic and deep feature extraction. A key advantage of this model lies in its use of an atrous spatial pyramid pooling (ASPP) module combined with an encoder–decoder structure, which enables the capture of rich semantic information at multiple scales while maintaining high sensitivity to detailed boundaries within the image. In this work, a DeepLabV3+ encoder pre-trained on a large-scale dataset served as the backbone network for feature extraction. This allows the model to adaptively learn discriminative feature maps, strongly correlated with the extrusion cycle count, from the complex textures, voids, cracks, and phase-separation characteristics present on the ABS fracture surfaces.

(4) Model Construction and Training

A classification model was constructed based on the aforementioned DeepLabV3+ network. Following the extraction of high-level features by the encoder, a global average pooling layer was used to transform the feature maps into a one-dimensional feature vector. This vector was then connected to fully connected layers and a Softmax output layer, culminating in a probability distribution output corresponding to the different extrusion cycle counts. The entire labeled image dataset consisted of 576 sub-images (256 × 256 pixels each), with 192 images corresponding to each extrusion cycle (1, 3, and 5). The dataset was randomly partitioned into training (80%, 460 images), validation (10%, 58 images), and test (10%, 58 images) sets. The validation set was used for hyperparameter tuning and early stopping, while the test set was held out for final evaluation of model performance. To further assess generalizability, additional images from two unseen ABS grades (0215H and HI121) were used as an independent cross-grade test set. During the training phase, the entire labeled image dataset was randomly partitioned into training and validation sets with a ratio of 8:2. The training process utilized the cross-entropy loss function, and the Adam optimizer was selected for iterative updates of the network weights. Model performance was evaluated by monitoring metrics such as classification accuracy, precision, recall, and macro-averaged F1-score on the validation set. Early stopping was implemented to prevent overfitting. Finally, the best-trained model underwent final evaluation on the independent test set, with its classification accuracy serving as the primary quantitative metric for assessing the model’s generalization capability.

## 3. Results and Discussion

### 3.1. Characterization of the Multiple-Extrusion 757K Microstructure

#### 3.1.1. FTIR

**Figure 3:**
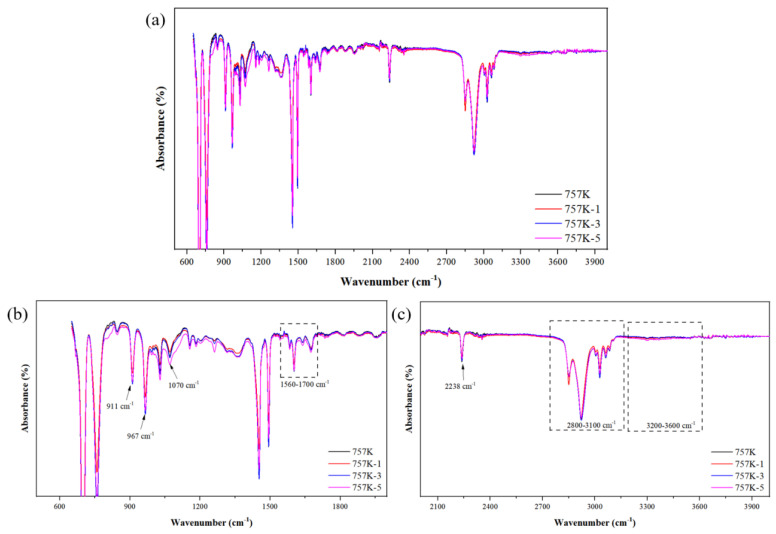
(**a**) FTIR spectra of 757K and multiple-extrusion 757K. (**b**) Local enlarged image at 400–2000 cm^−1^. (**c**) Local enlarged image at 2000–4000 cm^−1^.

Fourier-transform infrared (FTIR) spectroscopy was employed to analyze the initial molecular structure of the pristine ABS resin and to investigate its evolution through multiple-extrusion cycles. The FTIR results of 757K and multiple-extrusion 757K are shown in Figure 3. The degradation of ABS typically initiates with the destruction of the polybutadiene (PB) phase, generating free radicals. This is followed by the gradual formation of carboxylic acids, which subsequently affect the styrene-acrylonitrile (SAN) phase and the PB-SAN graft copolymer. Throughout this process, carbonyl, carboxyl, and ester groups are generated, leading to the gradual yellowing of the material.

In the FTIR spectra, characteristic peaks at 911 and 967 cm^−1^ are commonly used to quantify the PB phase. The absorption band at 1050–1100 cm^−1^ corresponds to ester groups formed from gelation products due to PB aging. The region between 1600 and 1700 cm^−1^ contains characteristic peaks for potential chromophoric groups such as carbonyl and carboxyl groups. The bands at 2800–3100 cm^−1^ are attributed to aliphatic C-H stretching vibrations, while the broad band at 3200–3600 cm^−1^ represents hydroxyl (O-H) groups. To obtain quantitative results, the peak at 2238 cm^−1^, corresponding to the C≡N stretching vibration of the acrylonitrile unit, was selected as an internal reference due to its stability and minimal involvement in oxidative degradation reactions.

The relative intensities of the characteristic peaks, normalized to the internal reference peak, are summarized in Table 1. As the number of extrusion cycles increased, the intensity of the peak at 967 cm^−1^, representing the PB phase, progressively decreased. Concurrently, the intensity of the peak at 1070 cm^−1^, assigned to ester groups from gelation products, showed a consistent increase, indicating the progressive destruction of the PB phase during processing. The absorption in the 1600–1800 cm^−1^ region, representing chromophoric groups, exhibited a gradual increase in intensity with more extrusion cycles, which is consistent with the observed yellowing of the samples. The intensity of the aliphatic C-H stretching vibration bands (2800–3100 cm^−1^) decreased with successive extrusions, suggesting chain scission events. Furthermore, the intensity of the broad hydroxyl band (3200–3600 cm^−1^) increased, confirming the formation of oxygen-containing groups (e.g., hydroperoxides, alcohols) as a result of thermo-oxidative degradation.

It is noteworthy that for the 757K grade, significant changes in these FTIR indicators became pronounced only after five extrusion cycles. In contrast, some domestic ABS grades show evident degradation after just one or three cycles. This indicates that the 757K resin possesses superior resistance to thermo-oxidative degradation, the underlying reasons for which will be explored in subsequent discussions.

These chemical changes in the PB phase and the formation of chromophores are the molecular origin of the morphological deterioration and yellowing discussed in the following sections.

#### 3.1.2. GPC

GPC was conducted to evaluate the changes in the molecular weight and molecular weight distribution of the SAN matrix within the ABS resin after multiple-extrusion cycles. The results, including the weight-average molecular weight (Mw), number-average molecular weight (Mn), and polydispersity index (PDI, calculated as Mw/Mn), are summarized in Table 2.

The data reveal that the molecular weight of the ABS resin remained nearly unchanged after the first extrusion cycle. A slight but consistent decrease in both Mw and Mn became apparent following the third and fifth extrusion passes. Specifically, after five cycles, Mw and Mn decreased by approximately 3.9% and 15.6%, respectively, compared to the virgin material. Concurrently, the PDI showed a gradual increase from 2.18 to 2.48, indicating a broadening of the molecular weight distribution.

These observations suggest that the SAN matrix possesses considerable stability during melt reprocessing. The minor reduction in molecular weight, which became significant only after multiple cycles, implies that chain scission events occurred at a relatively slow rate under the applied thermo-mechanical conditions. The increase in PDI suggests that the degradation is not perfectly random, potentially leading to a more heterogeneous chain length population. To ensure the reliability of these trends, the extrusion and GPC characterization were performed in two separate experimental runs, both of which yielded consistent results. The overall minimal change in molecular weight underscores the good reprocessing stability of this ABS grade, aligning with the FTIR observations that significant degradation manifested primarily after several extrusion cycles.

A closer examination of the molecular weight changes reveals a mechanistically significant pattern: after five extrusion cycles, Mn decreased by 15.6% (from 47,540 to 40,128) while Mw decreased by only 3.9% (from 103,574 to 99,520). This differential behavior indicates preferential chain scission of shorter chains or low-molecular-weight oligomers rather than random scission across all chain lengths. In a random scission process, both Mn and Mw would decrease proportionally, maintaining a relatively constant PDI. The observed larger relative drop in Mn suggests that the population of shorter chains is more susceptible to thermo-mechanical degradation, possibly due to their higher concentration of chain ends, which are known to be initiation sites for thermal oxidation and radical-mediated cleavage. Conversely, longer chains may be partially stabilized by entanglement effects or may experience slight cross-linking that counterbalances the mass loss from scission, leading to a less pronounced reduction in Mw.

The polydispersity index (PDI) increased from 2.18 (virgin) to 2.48 (after five cycles). Although formal statistical testing was not performed on the GPC data (as only single measurements per sample were conducted, albeit replicated in two independent experimental runs that yielded consistent trends), the monotonic and progressive increase across three extrusion levels (2.18 → 2.24 → 2.39 → 2.48) strongly suggests a genuine broadening of the molecular weight distribution rather than random variation. This broadening is consistent with the proposed mechanism: preferential scission of shorter chains generates an even larger population of very short chains (shifting the low-MW tail to lower values), while limited cross-linking or retention of longer chains maintains the high-MW tail, collectively widening the distribution.

The delayed reduction in molecular weight, together with the stable antioxidant content shown later by EDS, explains the retention of tensile strength observed in Section 3.2.2.

#### 3.1.3. SEM

To observe the phase morphology of ABS samples subjected to different numbers of extrusion cycles, SEM analysis was performed on the fracture surfaces obtained from the subsequent impact property tests, as will be described later. To reflect information regarding the uniformity of the phase morphology, two different locations were tested for each sample.

**Figure 4:**
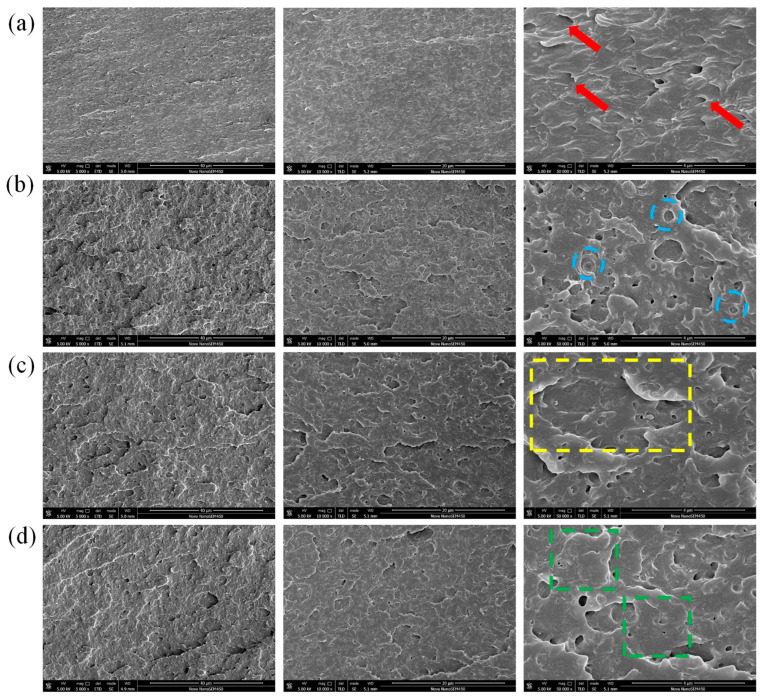
SEM images of (**a**) 757K, (**b**) 757K-1, (**c**) 757K-3, and (**d**) 757K-5.

The results indicate that the pristine sample exhibited excellent uniformity in its phase morphology distribution (Figure 4a). The impact fracture surface contained a multitude of dimples (marked by red arrows) of varying sizes and depths. These dimples are traces left behind by the PB phase particles, which initiate crazing and cavitation under impact stress and are ultimately stretched to rupture or debonded. The presence of dimples is direct evidence of the material’s ability to absorb a substantial amount of impact energy. This suggests that in the original ABS material, the PB toughening phase is well-dispersed, and the interfacial adhesion with the SAN continuous phase is strong, enabling effective craze initiation and termination, thereby conferring excellent impact toughness. Furthermore, the phase domain sizes at different locations in the 757K sample were highly uniform, indicating superb control over the consistency and homogeneity of its micro-phase morphology.

Significant changes in the material’s microstructure occurred with an increasing number of extrusion times. After one extrusion pass (Figure 4b), the fracture surface no longer displayed the typical, uniform, and deep dimple structure. Instead, it presented a porous, “fractured” or sponge-like morphology characterized by numerous irregular pores (marked by blue circles) of varying sizes and rough protrusions. This implies a fundamental shift in the material’s fracture behavior. When the number of extrusions increased to three (Figure 4c), this transformation became more pronounced. The irregular pore structures appeared to undergo a degree of coalescence and coarsening, forming larger, flake-like or plateau regions with rough edges (marked by yellow square). The interfacial morphology between the two phases or ligaments between pores appeared more fragile and loosely connected. The overall three-dimensional topography showed reduced relief compared to the once-extruded sample, beginning to exhibit a tendency towards brittle flattening.

After five extrusion times (Figure 4d), the deterioration in morphology intensified, and brittle characteristics became most evident. Larger, relatively flat, plate-like areas appeared, with further reduction in fine details such as small pores or fibrillar structures (marked by green square). Although porosity remained, the overall roughness and complexity of the topography decreased significantly, presenting a final morphology indicative of a transition from “ductile, porous fracture” to “quasi-cleavage brittle fracture.”

The SEM observations are highly consistent with the conclusions drawn from the aforementioned FTIR analysis. The FTIR spectra showed a continuous decrease in the intensity of the peak at 967 cm^−1^, characteristic of the PB phase, with increasing extrusion times, indicating chemical degradation of the PB phase. The SEM morphology visually reveals the specific manifestations of this chemical degradation at the microstructural level: reduction in rubber particle size, alteration of their shape, weakening of interfaces, and eventual structural breakdown. This progressive deterioration, from chemical structure to microscopic morphology, collectively constitutes the fundamental cause of the property degradation in ABS resin after multiple thermo-mechanical processing times.

This morphological transition from ductile dimples to brittle flats directly accounts for the severe loss of impact strength (48%) and ductility presented in Section 3.2.2.

#### 3.1.4. EDS

**Figure 5:**
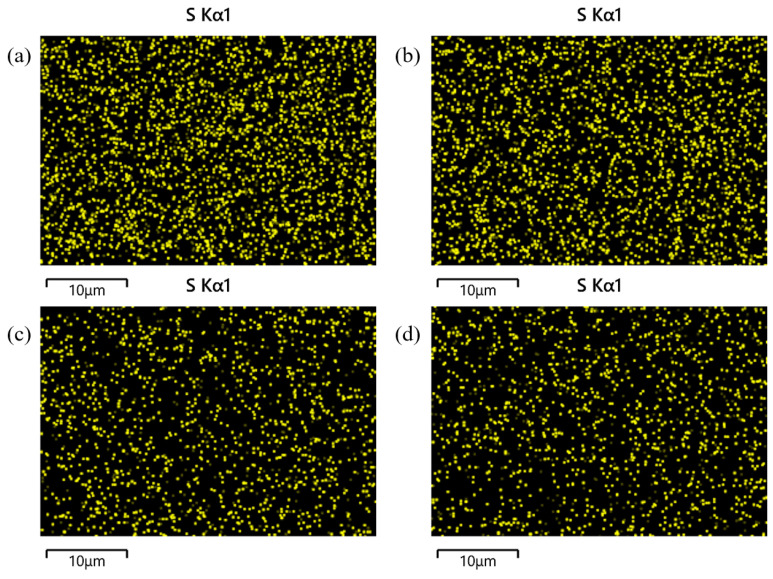
EDS spectra of 757K and multiple-extrusion 757K. Distribution of element S for (**a**) 757K, (**b**) 757K-1, (**c**) 757K-3, and (**d**) 757K-5.

To investigate the evolution of elemental composition during multiple-extrusion times, EDS analysis was performed on the impact-fractured surfaces, shown in Figure 5. The weight percentages of C, N, O, and S for the virgin and multiple-extrusion samples are summarized in Table 3.

Analysis of the data reveals distinct trends for each element. The C and N content shows minor fluctuations without a clear directional trend. The O content demonstrates a consistent and significant decline with increasing extrusion passes. This continuous loss suggests the volatilization of initial oxygen-containing components (e.g., residual monomers or additives) rather than significant post-processing oxidation, aligning with a thermally driven degradation mechanism dominant under the limited-oxygen extrusion conditions.

The behavior of S is of particular significance. It is critical to note that the ABS polymer matrix itself does not contain S. The detected S originates exclusively from sulfur-based antioxidant additives (e.g., thioester types) incorporated into the resin formulation. The EDS mapping of the S Kα1 signal for the virgin 757K sample (Figure 3a) shows a homogeneous dispersion of bright dots corresponding to the S signal, indicating a largely homogeneous distribution of the antioxidant within the SAN matrix, although minor local variations cannot be excluded due to the inherent resolution limits of EDS.

Remarkably, the quantitative data in Table 3 indicate that the S content does not exhibit a substantial or consistent decrease even after five extrusion times. The values remain within a relatively narrow range, and the measurements from two different locations for each sample are in close agreement. This demonstrates that the effective sulfur-based antioxidant components were not significantly consumed or degraded during the repeated thermo-mechanical processing and maintained a uniform distribution throughout the material.

This finding provides fundamental insight into the superior recycling stability observed for the 757K grade. The persistent presence and homogeneous dispersion of the sulfur-based antioxidant are decisive factors for its exceptional performance. These additives function by scavenging free radicals generated during processing, thereby effectively retarding the chain scission and cross-linking reactions that lead to molecular degradation. The stability of the S content directly correlates with the delayed onset of significant chemical changes observed via FTIR and the restrained molecular weight reduction seen in GPC, which collectively underpin the material’s retained mechanical properties, such as impact strength, over multiple processing cycles.

A closer examination of the oxygen content trend reveals an apparent counterintuitive phenomenon: the O wt% decreases consistently from approximately 3.9% (virgin) to 1.1% (after five cycles), rather than increasing as might be expected from oxidative degradation. This observation can be rationalized by considering the competing contributions of volatile oxygenated species and oxidative functionalization of the polymer backbone. Virgin ABS typically contains a certain amount of residual oxygen-containing low-molecular-weight compounds, including unreacted monomers (e.g., partially oxidized acrylonitrile), processing aids, stabilizer fragments, and oligomers bearing terminal hydroxyl or carbonyl groups. During each extrusion pass at 185–210 °C, these volatile species are progressively vaporized and removed from the system. While thermo-oxidative degradation does introduce new oxygen-containing groups (e.g., hydroperoxides, alcohols, ketones) onto the polymer chains via radical reactions, the mass gain from this surface-limited oxidation is relatively small and largely confined to the chain ends and amorphous regions. Furthermore, some of the newly formed oxygenated products may themselves be volatile or undergo further decomposition. The net result is a measurable decrease in total oxygen content as measured by EDS, which integrates all oxygen in the analyzed volume (approximately 1–2 μm in depth). This interpretation is fully consistent with the FTIR data, which showed normalized increases in hydroxyl and carbonyl peak intensities (Table 1)—those ratios reflect the concentration of oxygenated groups on the remaining polymer matrix, not the absolute oxygen mass. The stable sulfur content of the non-volatile antioxidant system further supports the volatilization argument, as sulfur-based additives are retained under the same processing conditions. Thus, the decreasing oxygen trend does not contradict the occurrence of polymer oxidation but rather highlights the complexity of mass balance in multiple-extrusion recycling.

The stable and uniform distribution of sulfur-based antioxidants is thus the key enabling factor for the delayed degradation kinetics observed in FTIR, GPC, and mechanical testing.

### 3.2. Characterization of 757K and Multiple-Extrusion 757K Properties

#### 3.2.1. DSC

**Figure 6:**
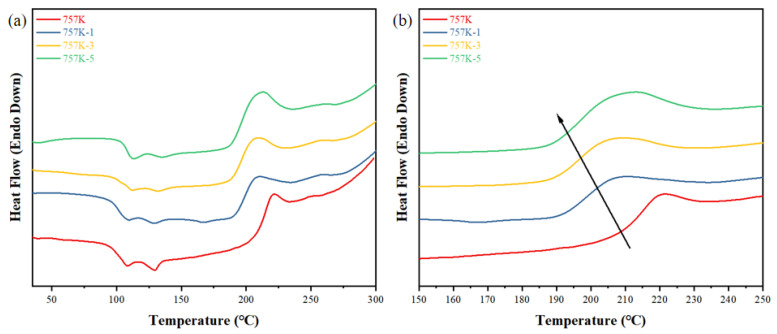
(**a**) Calorimetric curve of 757K and multiple-extrusion 757K. (**b**) Local enlarged image at 150–250 °C. The arrow shows the variation tendency of the thermal-oxidative degradation temperature.

To gain deeper insight into the thermal stability and oxidative degradation behavior of the ABS resin under repetitive processing, DSC was employed. The calorimetric curves for the 757K and multiple-extrusion 757K are presented in Figure 6a, with a detailed view of the primary transition region (150–250 °C) provided in Figure 6b.

A prominent, broad exothermic peak is observed for all samples within the temperature range of approximately 180 °C to 240 °C. Since ABS is an amorphous polymer with no melting or crystallization transitions in this temperature range, this exothermic event is unequivocally attributed to thermo-oxidative degradation of the polymer matrix during the non-isothermal DSC scan in air. Under these conditions, the unsaturated PB phase undergoes radical-mediated oxidation, including chain scission, hydrogen abstraction, and formation of oxygenated species, all of which are exothermic processes. Similar exothermic degradation peaks have been reported for ABS and other oxidizable polymers [33]. The onset temperature (T_onset) and peak temperature (T_peak) of this exotherm serve as quantitative indicators of the material’s resistance to thermal-oxidative degradation: lower values imply easier initiation and faster progression of oxidative reactions.

Quantitative analysis of the exothermic peaks shows that T_peak decreases progressively from 214 °C for virgin 757K to 206 °C after one extrusion, 201 °C after three extrusions, and 196 °C after five extrusions. T_onset follows a similar decreasing trend (from 198 °C to 182 °C). A systematic shift in this exothermic peak towards lower temperatures is evident with increasing extrusion cycles (Figure 6b). This trend provides direct evidence of the material’s declining thermal-oxidative stability upon repeated processing. The thermo-mechanical shear experienced during each extrusion cycle induces cumulative chain scission (as shown by GPC), generating more chain ends and weak links that serve as preferential initiation sites for oxidation. Consequently, the degraded material requires less thermal energy to initiate and sustain exothermic oxidative reactions, manifesting as lower T_onset and T_peak.

Furthermore, while the antioxidant system (indicated by stable sulfur content in EDS analysis) remains effective in retarding degradation, the progressive generation of new chain ends and potential weak links during each processing step gradually overwhelms its stabilizing capacity. The cumulative structural defects act as preferential sites for the initiation of oxidation, thereby lowering the overall activation energy for the thermal-oxidative process.

The progressive decrease in thermal-oxidative stability is a direct consequence of the accumulated chain scission (GPC) and PB phase damage (FTIR, SEM).

#### 3.2.2. Mechanical Properties

**Figure 7:**
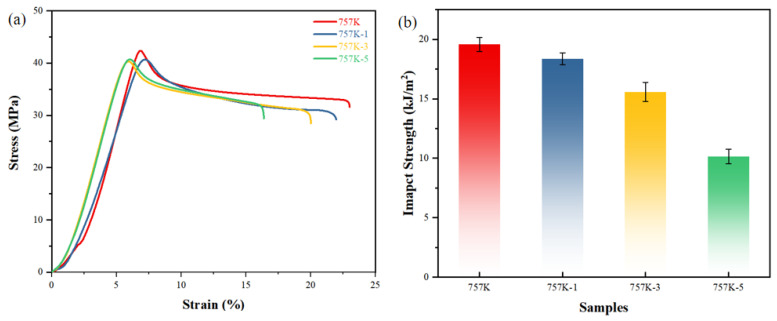
(**a**) Stress−strain curves and (**b**) impact strength of 757K and multiple-extrusion 757K.

The mechanical integrity of a polymer is paramount for its application and recyclability. To evaluate the effect of multiple-extrusion times on the mechanical performance of ABS 757K, tensile and impact tests were conducted. The stress–strain curves and the impact strength data are presented in Figure 7, with the corresponding quantitative tensile properties detailed in Table 4.

The tensile stress–strain curves (Figure 7a) exhibit the characteristic viscoelastic response of ABS. A clear trend is observed with increasing extrusion passes. The elastic (Young’s) modulus remains largely unchanged. However, the most pronounced effect is on the material’s ductility. The elongation at break undergoes a significant and monotonic decrease, dropping from 23.0% for the virgin ABS to 16.3% after five extrusions, representing a nearly 30% loss in ductility. Concurrently, the tensile strength at break shows a gradual decline from 42.4 MPa to 40.4 MPa.

This degradation in tensile performance, particularly the embrittlement evidenced by the reduced elongation at break, is a direct consequence of the chain scission events detailed in Section 3.1.2. The reduction in average molecular weight (Mw and Mn) weakens the chain entanglement network, which is critical for transmitting stress and enabling large plastic deformation. Shorter chains facilitate earlier disentanglement and crack initiation under load, leading to premature failure. The gradual drop in tensile strength further corroborates the loss of overall load-bearing capacity due to molecular degradation.

The impact strength demonstrates an even more dramatic decline (Figure 7b, Table 4). The impact strength decreases progressively from 19.6 kJ/m^2^ for the virgin ABS to 10.2 kJ/m^2^ after five extrusions, a reduction of approximately 48%. This severe embrittlement is the most sensitive mechanical response to the repeated processing history.

The drastic drop in impact strength can be mechanistically linked to the degradation of the PB phase, which is responsible for the energy-dissipating toughening mechanism in ABS. As supported by the FTIR and SEM observations, the PB phase undergoes chain scission and potentially cross-linking or oxidative hardening under thermo-mechanical stress. This degradation compromises the primary function of the PB particles, which is to cavitate and initiate massive shear yielding in the surrounding SAN matrix to absorb impact energy. As the PB particles lose their efficacy—either through size reduction, interface debonding, or chemical alteration—the crack initiation energy decreases, and cracks propagate more easily, leading to a brittle fracture mode. The correlation between the declining molecular weight, the deteriorating PB phase integrity, and the plummeting impact strength forms a coherent narrative of property degradation.

In summary, the mechanical property evolution paints a clear picture of cumulative damage. While the material retains a significant portion of its stiffness and strength even after five extrusion times, its toughness and ductility are severely compromised. This pattern is typical of the aging and reprocessing of thermoplastics, where chain scission preferentially erodes the properties dependent on long-range molecular interactions and energy dissipation mechanisms, rather than short-range bonding reflected in modulus. The stability of the antioxidant system, as discussed previously, may have moderated the rate of decline in tensile strength, but it could not prevent the catastrophic failure of the rubber toughening mechanism under impact, as evidenced by the steep drop in impact strength.

The embrittlement captured by mechanical testing mirrors the morphological changes in SEM and the molecular degradation in FTIR, and it correlates quantitatively with the yellowing progression shown next.

#### 3.2.3. Yellow Index (YI)

**Figure 8:**
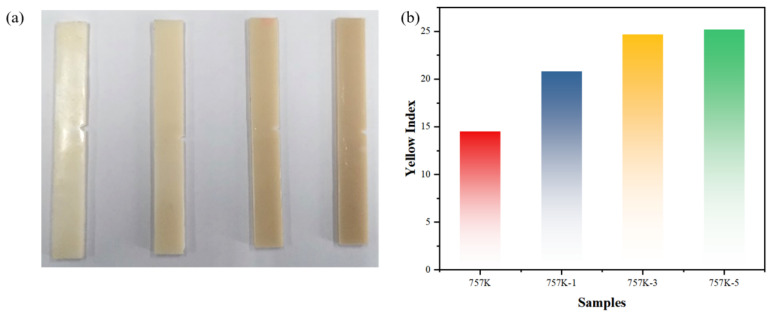
(**a**) The photo shows 757K, 757K-1, 757K-3 and 757K-5 sequentially from left to right and (**b**) the yellow index of 757K and multiple-extrusion 757K.

A pronounced and systematic change in the sample’s color is evident from the photographs in Figure 8a. The virgin 757K sample exhibits a near-translucent, milky-white appearance. With each successive extrusion pass, the sample color progressively darkens to a distinct yellowish hue. The 757K-5 sample displays a clear tan or light brown coloration. This visual transition provides immediate, qualitative evidence of cumulative chemical changes induced by thermo-mechanical processing.

This visual assessment is conclusively quantified by the corresponding yellow index (YI) measurements presented in Figure 8b. The YI value increases monotonically and almost linearly with the number of extrusion times. The virgin material possesses the lowest YI. A significant jump is observed after the first extrusion, and the YI continues to rise steadily, reaching its maximum for the 757K-5 sample.

The observed yellowing is a classic signature of polymer oxidation, specifically within the PB phase of ABS. The C=C in the PB chains is highly susceptible to thermal-oxidative attack during high-temperature processing, even in the presence of antioxidants. The mechanism involves the formation of hydroperoxides followed by their decomposition, leading to the generation of various chromophores. These include carbonyl groups and, more importantly, conjugated polyene sequences formed through chain scission and rearrangement reactions. These conjugated structures are strong absorbers of visible light approximately 400–450 nm, causing the reflected or transmitted light to appear yellow to brown.

The progressive increase in YI with each processing time indicates that the antioxidant system, while effective in stabilizing molecular weight to a degree, cannot completely prevent the formation of these colored oxidation products. Each extrusion pass subjects the material to another cycle of thermal and shear stress, causing incremental oxidative damage that cumulatively manifests as increased yellowness. This trend aligns with the declining onset temperature for thermal-oxidative degradation observed in Section 3.2.1, as both phenomena share the same root cause: the progressive oxidation of the polymer. Furthermore, the yellowing serves as a macro-scale indicator correlating with other property declines. The formation of chromophores and the associated chain scission within the PB phase directly contribute to the embrittlement of the PB particles, which is a primary reason for the severe deterioration in impact strength. Thus, the YI is not merely a cosmetic metric but a non-destructive, quantitative measure that reflects the underlying chemical degradation responsible for mechanical failure.

Thus, YI serves as a non-destructive proxy for the chemical degradation that also drives mechanical property loss, and its monotonic trend provides a simple macroscopic validation of the cumulative aging effect.

#### 3.2.4. Melt Flow Rate (MFR)

The melt flow index (MFI), measured under different loads, and the derived melt flow ratio (MFR) provide key insights into the viscoelastic properties and structural evolution of ABS 757K after multiple extrusion. The relevant data are summarized in Table 5.

The melt flow index shows a certain degree of decrease after multiple extrusions. One possible explanation is that the multiple-extrusion process may induce a certain level of cross-linking in the PB phase, potentially accompanied by the formation of gel-like substances. The increased ester peak observed in FTIR (Table 1) could be consistent with such structural changes, although alternative interpretations cannot be ruled out. If cross-linking occurs, it will contribute to a three-dimensional network that hinders macromolecular flow, thereby reducing the melt index. However, direct evidence is still needed to confirm this hypothesis. At the same time, the MFR did not change significantly. This is because although the reduction in the molecular weight would theoretically improve flowability, the combined effects of partial cross-linking in the PB phase, potential formation of gel-like substances, and the loss of small molecules and additives in the resin may have collectively contributed to the observed changes in melt flowability. Definitive assignment of the dominant mechanism requires further investigation using rheological characterization and gel content analysis.

The complex melt flow behavior, influenced by both chain scission and possible slight cross-linking, adds a rheological dimension to the multi-scale degradation picture and underscores that no single parameter captures the full aging state—motivating the image-based holistic predictor presented in Section 3.3.

### 3.3. Aging Degree Prediction via Image Recognition

Building upon the comprehensive characterization of physicochemical degradation, this study further explores a novel, non-destructive approach to quantify the aging degree of recycled ABS. A deep learning model based on the DeepLabV3+ architecture was developed to predict the number of extrusion cycles directly from the microscopic morphology of impact-fractured surfaces. The performance and generalizability of the model are evaluated and discussed herein.

**Figure 9:**
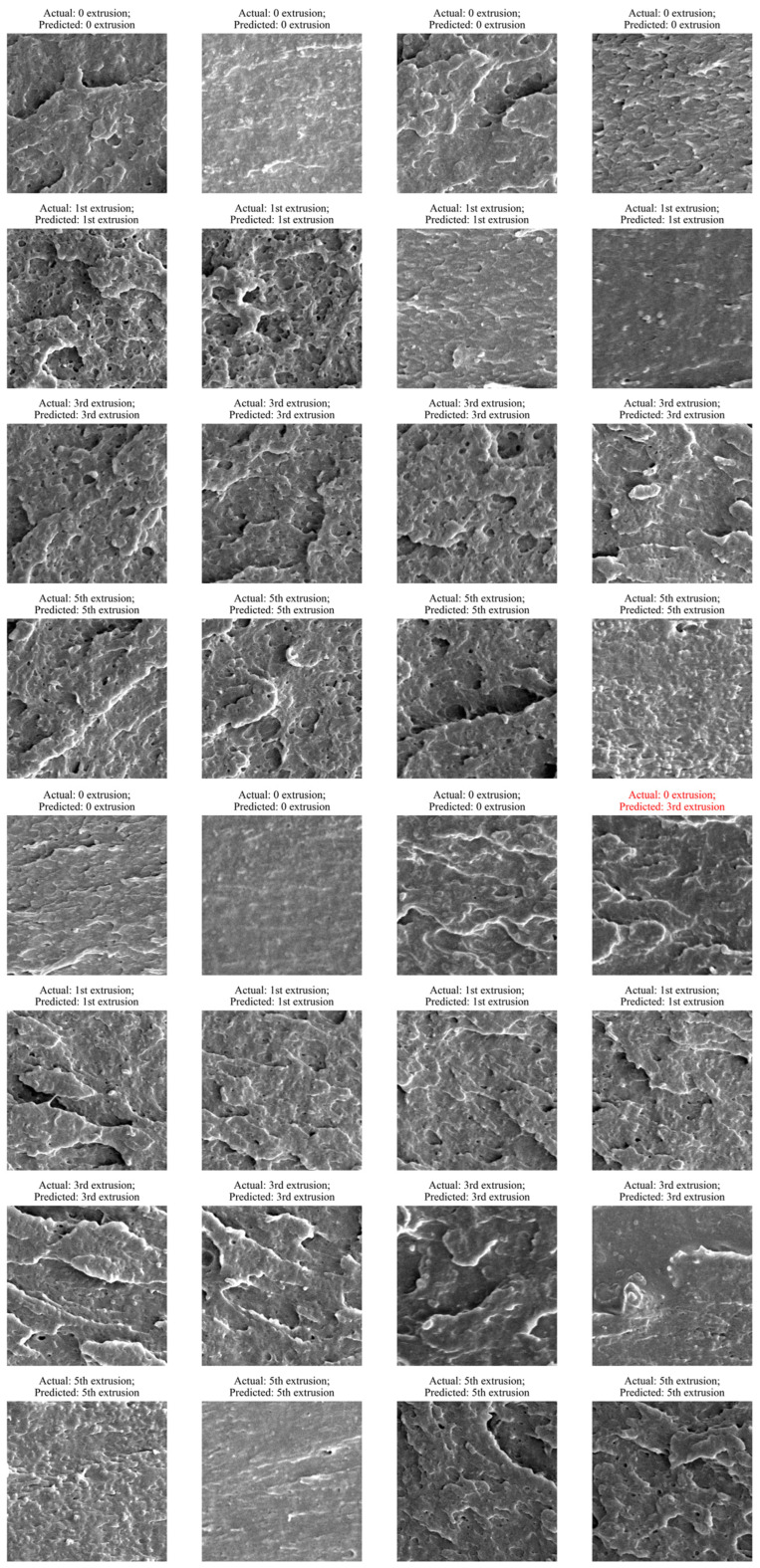
Representative results of aging degree prediction via image recognition. Each panel displays an SEM image of an impact-fractured surface. The actual number of extrusion cycles (‘Actual: X’) and the model’s prediction (‘Predicted: X’) are both labeled directly on the same image.

The model’s performance is qualitatively illustrated in Figure 9. Each panel compares the SEM image of a fracture surface with its actual model and predicted extrusion time. The figure effectively showcases the model’s high success rate, as the predicted labels match the true labels in the vast majority of cases. This visual evidence confirms that the model has successfully learned to correlate subtle, human-eye-indistinguishable textural and topological features in the grayscale SEM images with the precise number of extrusion cycles.

While the model achieves high overall accuracy, a small fraction of misclassifications (typically 1–2 images per 58-test set) was observed. Examination of these misclassified images revealed that they correspond to fracture surfaces exhibiting morphologically intermediate or locally heterogeneous features. For instance, a 3-cycle sample that locally retains dimple-like ductile features (resembling 1-cycle morphology) or shows premature brittle flattening (akin to 5-cycle morphology) may be mispredicted as an adjacent cycle. Such ambiguity likely arises from non-uniform degradation across the sample cross-section or local variations in antioxidant distribution. Encouragingly, misclassifications between non-adjacent cycles never occurred, indicating that the model robustly captures the overall degradation trend.

**Figure 10:**
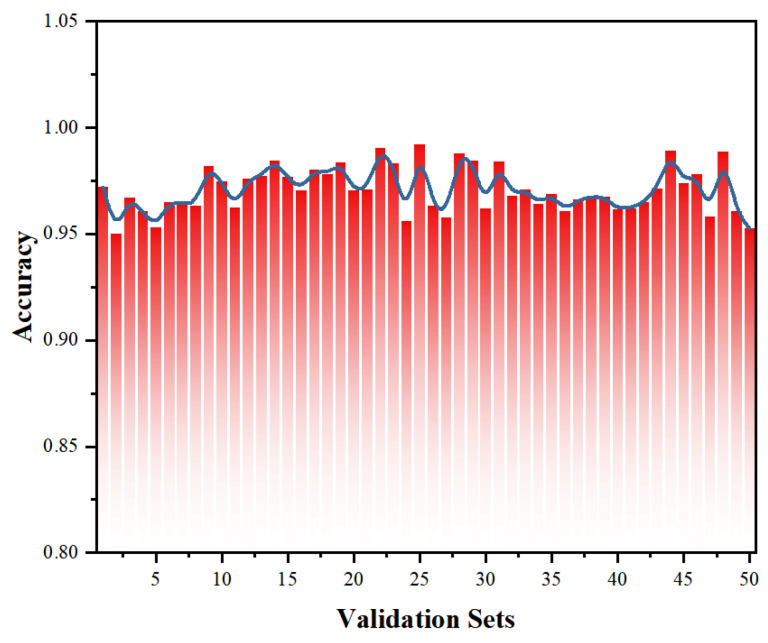
The accuracy of the validation sets.

The trained model demonstrated a high degree of accuracy in classifying the extrusion history of ABS samples. The primary quantitative result, as shown in Figure 10, is the validation accuracy across 50 independent validation sets. The accuracy consistently remains above 0.95, with an average exceeding 96.5%. This indicates a robust and reliable predictive capability of the model. Crucially, the validation sets were not limited to the 757K grade used for training. Samples from two other commercially prevalent ABS grades in China, 0215H and HI121, which possess different formulations and baseline properties, were also included in the validation. The model maintained an accuracy of approximately 96.5% on these unseen grades. This exceptional cross-grade generalizability is a significant finding, suggesting that the microstructural features indicative of thermo-mechanical aging (e.g., changes in PB particle morphology, matrix texture, and fracture surface roughness) learned by the model are fundamental and transferable across different ABS formulations. It implies that the degradation patterns induced by repeated extrusion, while varying in absolute rate, follow a common morphological evolution pathway that the convolutional neural network (CNN) can effectively detect and quantify.

The success of this image recognition approach establishes a powerful, rapid, and minimally invasive tool for assessing the history and state of recycled ABS. Traditional methods for characterizing degradation (e.g., GPC, FTIR, mechanical testing) are time-consuming, destructive, and require specialized equipment and operators. In contrast, once trained, the deep learning model can provide an instant prediction of the extrusion history from a single SEM image, which could potentially be adapted for faster, lower-resolution imaging techniques. The 96.5% accuracy across multiple grades underscores its practical utility in recycling streams where plastics of varying grades are mixed. The method directly links the macroscopic, engineering-relevant parameter (“number of times recycled”) to the fundamental microstructural changes, providing a new paradigm for quality control and lifecycle assessment of recycled polymers.

In summary, the deep learning model successfully predicts the extrusion cycles of ABS resins with high accuracy (>96%) based solely on fracture surface morphology. Its performance on unseen resin grades (0215H, HI121) confirms the generalizability of the learned degradation features. This work validates image recognition as a potent and efficient tool for quantifying polymer aging, bridging advanced machine learning with materials science to address practical challenges in polymer recycling.

### 3.4. Discussion

The systematic investigation of ABS 757K subjected to up to five extrusion times has provided a multi-faceted and coherent understanding of its degradation mechanism. The integration of chemical, morphological, thermal, mechanical, and rheological data reveals a consistent narrative of cumulative, thermo-mechanically induced damage, while the successful application of deep learning offers a novel paradigm for its assessment.

The logical chain connecting the experimental observations can be summarized as follows: The persistent efficacy of sulfur-based antioxidants (EDS) delays the onset of significant chain scission (GPC) and PB phase oxidation (FTIR). However, cumulative thermo-mechanical stress eventually leads to PB degradation, forming chromophores (yellowing, YI) and altering the rubber particle morphology (SEM). This microstructural breakdown directly causes embrittlement (severe drop in impact strength and elongation at break). Concurrently, the accumulated defects lower the activation energy for thermal-oxidative degradation (DSC), while changes in molecular architecture (chain scission vs. possible cross-linking) manifest in altered melt flow (MFR). Ultimately, all these molecular-to-macroscopic alterations leave a distinct fingerprint on the impact fracture surface, which the deep learning model learns to decode—thus the image recognition approach provides an integrated prediction of the ‘aging degree’ that no single characterization method alone could offer.

(1) Superior Stability of ABS 757K and the Role of Antioxidants

A pivotal finding of this work is the identification of the key factor behind the relatively superior recycling stability of the 757K grade compared to other commercial ABS resins. While chain scission (evidenced by GPC), PB phase degradation (indicated by FTIR and SEM), and resultant embrittlement (shown by mechanical testing) occurred progressively, the onset of significant deterioration was delayed. The EDS analysis provided the critical link: the S content, originating from sulfur-based antioxidants, remained remarkably stable and uniformly distributed even after five extrusion times. The counterintuitive decrease in oxygen content with progressive extrusion cycles, explained by the volatilization of residual oxygenated small molecules, underscores the importance of considering mass balance alongside chemical functionalization when interpreting EDS data in recycled polymers. This demonstrates that the antioxidant package was neither rapidly consumed nor thermally destroyed under the applied processing conditions. Its persistent efficacy in scavenging free radicals effectively retarded the autocatalytic oxidative degradation pathway, thereby slowing down the rate of molecular weight reduction and the chemical transformation of the PB phase. This fundamental insight explains why properties like tensile strength and modulus were largely preserved until later cycles, whereas the more sensitive impact strength, heavily dependent on the integrity of the rubber phase, showed an earlier and more dramatic decline.

(2) A Multi-Scale Degradation Mechanism

The degradation follows a cascade of events across multiple length scales. At the molecular level, the primary event is the thermo-mechanical chain scission within both the SAN matrix and, more critically, the PB phase. This is confirmed by the reduction in Mn and Mw and the decrease in the characteristic PB FTIR peak intensity. Chain scission creates more chain ends, lowers the entanglement density, and generates macro-radicals. In the presence of trace oxygen, these radicals lead to the formation of oxygenated groups (hydroxyl, carbonyl, carboxyl), as seen in FTIR. For the PB phase, this oxidation is particularly consequential. It not only severs the chains but also leads to the formation of conjugated polyene sequences, which are direct chromophores responsible for the linear increase in yellow index (YI). Concurrently, it is plausible that limited cross-linking or the formation of gel-like products may occur, as suggested by the rising ester peak in FTIR and the reduced melt flow index. However, alternative interpretations (e.g., chain scission leading to broadened molecular weight distribution and altered entanglement density) could also explain some of the rheological changes. Future work employing dynamic rheometry and gel fraction measurements is required to conclusively determine whether cross-linking or gelation indeed takes place during multiple extrusion of ABS.

At the micro-scale, the chemical degradation of the PB phase manifests as physical deterioration. SEM analysis visually captured the transition from a ductile, dimple-dominated fracture surface to a brittle morphology. The degradation of PB particles compromises their ability to cavitate and initiate shear yielding. The particles may become smaller, harder, and debond more easily from the SAN matrix. This microstructural breakdown directly translates to the catastrophic loss in impact toughness, as the primary energy-absorption mechanism is rendered ineffective.

The macro-scale properties are the direct consequence of these underlying changes. The reduced molecular weight and weakened PB-SAN interface led to embrittlement. The thermal-oxidative stability, as probed by DSC, decreases because the accumulated structural defects serve as preferential initiation sites for exothermic oxidation, lowering the required activation energy.

(3) Synergy Between Characterization Techniques and the Image Recognition Breakthrough

The deep learning model successfully distills this complex, multi-parameter degradation state into a single, image-based predictor. The model’s high accuracy (>96.5%) in predicting extrusion cycles, even for unseen ABS grades (0215H, HI121), is a significant breakthrough. It empirically proves that the sequential micro-morphological evolution captured by SEM—from ductile dimples to brittle flats—is a universal and reliable indicator of thermo-mechanical aging in ABS, transcending specific formulation differences. The model effectively quantifies the “aging degree” by recognizing these subtle, hierarchical texture patterns that are beyond human visual discrimination. This bridges the gap between fundamental microstructural science and applied industrial need, offering a rapid, non-destructive tool for quality assessment in recycling streams.

(4) Limitations and Future Outlook

While this study establishes a clear mechanism and a novel predictive tool, some questions remain. The exact nature of the minor cross-linking/gelation suggested by rheology and FTIR warrants further investigation using techniques like rheometry. Furthermore, the model’s performance on resins with vastly different stabilizer packages or blended with other polymers should be tested. Future work could focus on expanding the image database to include more grades and processing conditions, refining the model for regression (predicting continuous property values rather than discrete cycles), and exploring the use of faster, more accessible imaging techniques to enhance practical applicability. While the SEM images presented here clearly show the morphological trend, higher-resolution techniques such as atomic force microscopy (AFM) or focused ion beam-scanning electron microscopy (FIB-SEM) could provide quantitative surface roughness and three-dimensional topography data in future studies.

## 4. Conclusions

This work has comprehensively elucidated the structure-property evolution of ABS resin during multiple-extrusion cycles and demonstrated a highly effective image-recognition-based method for predicting the aging degree. The progressive thermo-oxidative degradation of ABS 757K upon repeated extrusion is characterized by chain scission (evidenced by reduced Mn and Mw), oxidation of the PB phase (formation of carbonyl/hydroxyl groups and conjugated chromophores), and consequent deterioration of the PB phase morphology, which transitions from ductile dimple-dominated fracture surfaces to brittle, quasi-cleavage morphologies.

The superior retention of properties during the initial extrusion cycles for the 757K grade is attributed to the exceptional stability and homogeneous distribution of its sulfur-based antioxidant system, as directly revealed by EDS mapping. These antioxidants function as effective free radical scavengers, thereby retarding the degradation kinetics and delaying significant molecular weight reduction until later processing cycles. The macro-scale property deterioration is a direct consequence of these microstructural changes: the reduction in impact strength (48%) and elongation at break (30%) is primarily driven by the chemical and physical degradation of the PB phase, while the complex changes in melt flow behavior may arise from combined chain scission and possible slight cross-linking, though the latter requires further validation.

A deep learning model based on the DeepLabV3+ architecture was successfully developed to predict the number of extrusion cycles directly from SEM images of impact fracture surfaces. The model achieved an average accuracy exceeding 96.5% and, remarkably, demonstrated excellent cross-grade generalizability, maintaining high accuracy on two completely unseen commercial ABS grades (0215H and HI121). This finding proves that the micro-morphological evolution pathway is a fundamental and transferable fingerprint of thermo-mechanical aging in ABS.

Collectively, this research provides a dual contribution: a detailed mechanistic framework linking multi-scale structural degradation to macro-property losses in recycled ABS, and a novel, rapid, non-destructive predictive tool based on image recognition. The proposed technology offers a practical, intelligent solution for real-time quality control and lifecycle assessment in plastic recycling streams, enabling operators to instantly assess the degradation state of post-consumer ABS from fracture surface images alone—thereby advancing the vision of a smart circular economy for engineering thermoplastics. Furthermore, while the current model performs discrete classification (predicting the number of extrusion cycles), future work should explore regression-based approaches to predict a continuous ‘aging degree’ index. This would enable finer resolution and provide a more nuanced assessment of partially degraded materials in recycling streams.

## Figures and Tables

**Table 1:** Relative intensity of characteristic FTIR peaks (normalized to the internal reference peak at 2238 cm^−1^) for 757K and multiple-extrusion 757K samples.

Samples	Characteristic Peaks (cm^−1^)				
	967	1070	1600–1800	2800–3100	3200–3600
757K	2.729	1.131	2.090	14.838	2.216
757K-1	2.717	1.230	2.132	14.707	2.677
757K-3	2.638	1.241	2.172	14.347	2.939
757K-5	2.398	1.321	2.365	12.538	3.337

**Table 2:** Molecular weight and Polydispersity Index of 757K and multiple-extrusion 757K.

Samples	Mw	Mn	PD
757K	103,574	47,540	2.18
757K-1	103,010	46,080	2.24
757K-3	100,699	42,096	2.39
757K-5	99,520	40,128	2.48

**Table 3:** Results of EDS element content percentage of 757K and multiple-extrusion 757K.

Elements	757K		757K-1		757K-3		757K-5	
	Side 1	Side 2	Side 1	Side 2	Side 1	Side 2	Side 1	Side 2
C	88.71	87.32	89.45	89.96	90.35	90.70	89.76	91.26
N	6.24	6.85	5.81	5.70	5.75	5.73	5.92	5.92
O	3.99	3.85	2.35	2.21	1.96	1.10	1.09	1.13
S	2.07	2.10	2.12	1.93	1.75	1.88	1.54	1.65

**Table 4:** Tensile properties of 757K and multiple-extrusion 757K.

Samples	Elastic Modulus (MPa)	Elongation at Break (%)	Tensile Strength (MPa)	Impact Strength (kJ/m^2^)
757K	968.3 ± 19.4	23.0 ± 2.9	42.4 ± 0.3	19.6 ± 1.0
757K-1	1011.3 ± 20.2	22.2 ± 2.4	41.1 ± 0.6	18.4 ± 0.9
757K-3	1014.4 ± 20.3	20.0 ± 2.3	40.6 ± 0.4	15.6 ± 0.8
757K-5	1008.8 ± 20.2	16.3 ± 2.1	40.4 ± 0.8	10.2 ± 0.5

**Table 5:** Melt Index and MFR of 757K and multiple-extrusion 757K.

Samples	Melt Index, g/10 min, 220 °C		MFR
	2.16 kg	5 kg	
757K	1.67	5.21	3.12
757K-1	1.58	5.16	3.26
757K-3	1.45	5.14	3.54
757K-5	1.45	4.75	3.28

## Data Availability

The data are not publicly available due to confidentiality restrictions related to this ongoing research.
